# Fabrication of Self-Assembling Carbon Nanotube Forest Fishnet Metamaterials

**DOI:** 10.3390/nano12030464

**Published:** 2022-01-28

**Authors:** Adam Pander, Takatsugu Onishi, Akimitsu Hatta, Hiroshi Furuta

**Affiliations:** 1 Electronic and Photonic Systems Engineering, Department of Engineering, Graduate School of Engineering, Kochi University of Technology, Tosayamada, Kami City 782-8502, Kochi, Japan; onishi.takatsugu@gmail.com (T.O.); hatta.akimitsu@kochi-tech.ac.jp (A.H.); 2 Center for Nanotechnology, Research Institute, Kochi University of Technology, Tosayamada, Kami City 782-8502, Kochi, Japan

**Keywords:** carbon nanotubes, fishnet, metamaterial, blue shift, self-organization, large scale, colloidal lithography

## Abstract

The investigation of the preparation of polystyrene (PS) nanosphere monolayers for the fabrication of carbon nanotube (CNT) forest fishnet metamaterial structures is studied in this paper, as a cheap alternative for top-down patterning methods. The precise control of dry etching conditions resulted in a highly controlled diameter of PS nanobeads, which were then used as a shadow mask for CNT fishnet preparation. The change of the size of the holes from 370 nm to 665 nm resulted in a gradual change of the CNT morphology from multi-walled to single-walled CNTs. The ultraviolet-visible (UV-Vis) reflectance spectra showed that the variation of the hole diameter resulted in the nonlinear light absorption in CNT fishnets that caused the change of the resonance frequency. The change of the fishnet wire width (inductance) and the hole size (capacitance) resulted in the blueshift of the broadband resonance frequency peak. The presented work has a significant potential to allow for the large-scale fabrication of CNT-based fishnet metamaterial structures for applications in energy harvesting, energy storage, solar cells, or optoelectronic devices, such as neuromorphic networks.

## 1. Introduction

Metamaterials are artificially designed media to exhibit properties that are yet to be encountered in natural materials. The structure of metamaterials is composed of the so-called meta-atoms, which are subwavelength structures grouped together, to resemble the atomic lattice of natural materials, in order to exhibit the required values of permeability and permittivity in the desired frequency range [1,2,3]. To date, the majority of metamaterials have been designed based on a simple inductor–capacitor (LC) circuit, in a split-ring resonator (SRR) or asymmetric SRR (ASR) geometry [4,5]. To interact with electromagnetic (EM) radiation at different frequencies, the control of the size, shape of meta-atoms, and the material of these structures is required. However, the increase in the operating frequency requires the scaling down process, which usually results in the introduction of the inner material defects to resonator unit cells. Therefore, in recent years, some novel approaches have been investigated, and various nonlinear media were studied [6,7], among which carbon nanotubes (CNTs) were also investigated, as they possess very distinctive properties.

The unique physical structure of CNTs [8,9] and their optical and electric properties [10,11,12] has led to an extraordinary behavior, such as anisotropic optical absorption [13] and anisotropic electrical conductivity of aligned CNT forest [14,15]. Moreover, in the external EM field, CNTs behave as almost ideal one-dimensional nanorod antennas with a diameter of a few nanometers and length up to hundreds of micrometers, while single-walled CNTs (SWNTs) exhibit behavior similar to direct gap semiconductors with absorption spectra dominated by exciton lines [16]. The experimental observations of CNTs showed that the nonlinear behavior is related to a high third-order susceptibility with a picosecond recovery rate, which has been utilized in various promising applications, such as nanoscale light sources, photovoltaic devices, lasers, and recently also in various metamaterial designs [17,18,19]. Reports regarding the combination of metamaterial and photonic design with CNTs were presented by several groups. Butt et al. [20,21] showed photonic devices fabricated as the arrays of multi-walled CNTs (MWNTs) for plasmonic waveguides [21,22], while Hong et al. [23] studied terahertz metamaterials composed of CNT films with various sizes of slits to interact with the terahertz waves. The spray-coated CNT films prepared on Si_3_N_4_ ceramic metamaterials were also presented to improve the general performance of the metamaterial composite in the EM field [12]. Recently, CNT composite structures have been also investigated, showing a negative permittivity behavior [24] and high radiation absorption [25] at various frequencies. Moreover, our group presented the first fabrication of SSR structures from the CNT forest and investigated the influence of the geometry and the inner structure on their reflectance in the near-infrared (NIR) regime [26,27], with potential use for energy harvesting and storage, absorbers, and also other optoelectronics devices, such as neuromorphic networks. Despite very promising results and very interesting properties, all of the fabrication methods in the above mentioned works were based on the top-down patterning approach, which despite providing high-quality structures, is generally time consuming, has relatively low cost-effectiveness, and from which it is difficult to obtain large-scale structures.

To achieve small size unit cells in large-scale environments, the self-assembly methods have been investigated. Some of them used metallodielectric materials out of SrTiO_3_–TiO_2_ eutectic composite to obtain SRR microstructures [28], while in other works, the synthesis of chiral meta-atoms was achieved by organic self-assembly from various template materials [29]. The fabrication of plasmonic metamaterials in the form of fishnets was also presented based on template annealing, or colloidal crystal templates as shadow masks for deposition (called also colloidal lithography) [30,31,32,33,34,35], which includes silica nanoparticles, polystyrene (PS) nanospheres, etc. Recently, double-layer fishnet metamaterials, electromagnetic composites, or fishnet-like metallic structures have been vastly studied in various applications, due to their high spectral absorption performance, as broadband negative index materials for absorbers [36] or invisibility cloaks [37]. Furthermore, the quasi-optical devices based on fishnet structures, such as polarizers, lenses, or demultiplexers have also been presented at millimeter wave bands [38,39,40,41].

At the same time, the self-assembly methods for the fabrication of various CNT-based structures were also investigated. Wang et al. [42] showed the self-organization of SWNTs into rings, membranes, and even nano letters, based on the patterned chemical templates and SWNT assembly from solutions. Other methods of the CNT assembly were also presented and include the Langmuir–Blodgett technique [43], external field-assisted routes [44,45,46,47], electrospinning [48], and transfer printing [49]. However, none of these methods allows for a large-scale fabrication at the same time, limiting the self-organized structures mostly to a micrometer, sometimes millimeter scale. Thus, in this work, we plan to explore the bottom-up approach for self-organizing materials, which could assist in the future development of low-cost and large-scale CNT metamaterials.

One of the possible candidates for large-scale structures is a CNT forest fishnet metamaterial that can be fabricated using the existing methods, which showed a very good performance in the fabrication of previously described fishnet designs. Periodic CNT fishnets could be efficiently used in energy harvesting and storage [50,51], or solar thermophotovoltaic devices [52]. In these applications, the light trapping abilities of vertically-aligned CNTs can be modified and precisely controlled by the geometry of the fishnet structure, such as hole diameter and lattice period size, similar to random CNT-based honeycombs [51,53]. In CNT fishnets, the control of the surface area can be achieved to enhance light absorption at different wavelengths, allowing also for absorption selectivity at the same time. Furthermore, the combination of CNT fishnet structures with other materials, such as polymers or dielectrics, allows the formation of composites with the same electromagnetic properties and a very high mechanical strength for rheological [54,55,56], flexible electronic [50], and even supercapacitor applications [57]. Therefore, the purpose of this study is to investigate the preparation methods and structural dependence of the fabricated structures on the optical properties of self-assembling CNT forest fishnets [58]. In this work, the novel self-organized structure of CNT forest fishnets is presented. Throughout this work, the templates based on polystyrene (PS) nanospheres are used as a mask during the catalyst deposition, before the growth of CNTs. The demonstration of CNT fishnets with different hole sizes is presented and the nonlinear optical properties of these structures are discussed with consideration of future applications.

## 2. Materials and Methods

The fabrication of the CNT forest fishnet structures was carried out as follows: (1) self-organization of PS nanobeads into a monolayer on the substrate, (2) shrinking of PS nanobeads by reactive ion plasma etching, which was followed by (3) the deposition of AlO*_x_*/Fe catalyst, (4) nanobead removal, and (5) chemical vapor deposition (CVD) synthesis of CNT forests in the fishnet areas.

**Figure 1:**
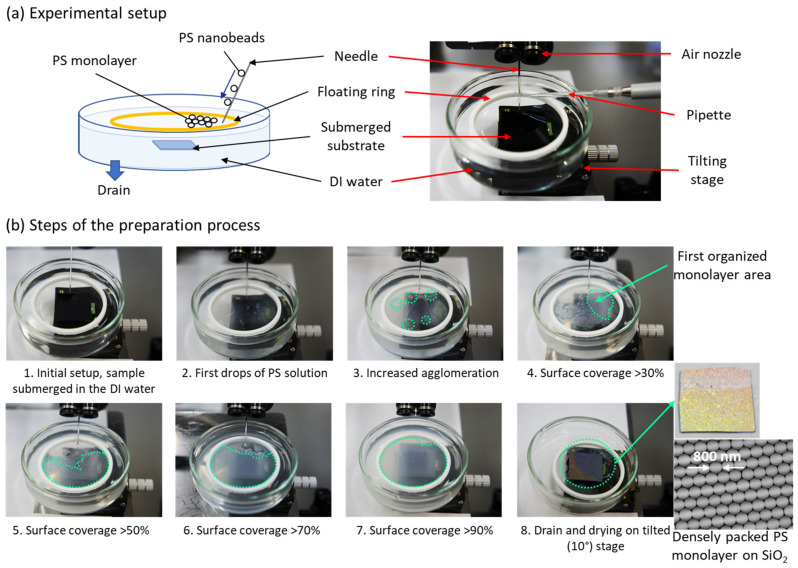
(**a**) Schematic and photo of the experimental setup used for the preparation of a monolayer of PS nanobeads on the surface of SiO_2_ substrate. (**b**) The steps of the monolayer preparation process using 800 nm diameter PS nanospheres.

In Figure 1a, the schematic and photo of the experimental setup are shown. The assembly of a monolayer of PS nanobeads was carried out according to the procedure shown in Figure 1b. First, an electrochemically polished (R_a_ < 0.15 nm) th-SiO_2_ substrate was placed at the bottom of a clean Petri dish, which was then filled with deionized (DI) water. Then, a polytetrafluoroethylene (PTFE) ring with a diameter of 5 cm was carefully placed on the water surface, serving as area confinement for PS nanospheres. The PS nanobead DI water suspension was purchased from Sigma-Aldrich (Tokyo, Japan) with an average size of spheres of 0.8 μm. The PS nanobead suspension was mixed with ethanol before use at various ratios, from 1:1 to 1:4, and then applied slowly to the water surface by a pipette. To avoid turbulence and significant agglomeration of the nanobeads on the surface, the application of the PS solution by pipette was carried out using a needle, which worked as the conduct of the solution to the water surface, allowing for homogenous spread. The solution was slowly applied until the area within the PTFE ring was occupied by PS nanobeads in about 90–95%. After a few hours, a high-quality monolayer was formed at the water–air interface. It should be noted that an additional airflow from the directed nozzle was used to enhance the formation of a large-area self-oriented surface. The PS monolayer was transferred onto the SiO_2_ substrate by slowly draining the water, which after drying off from the surface, allowed to keep the original packing structure of nanobeads.

To ensure the fabrication of a highly packed PS monolayer film, the precise control of the speed and method of the application process of the PS colloidal solution to the water–air interface is required. Generally, a lower and constant dosage speed allows the avoidance of possible turbulences and reduces the formation of PS agglomerates. The additional airflow from the nozzle, directed to the surface, pushes the nanobeads on the water surface towards the walls of confinement space and allows for more homogenous spread. The ratio of the initial colloidal solution with the solvent should be tuned to allow for a good flow of the PS nanobeads during the applications process. Finally, to avoid introducing additional turbulence to the already formed monolayer, the draining of the water should be carried out from the bottom of the cuvette, at a relatively slow speed. 

The transfer of PS monolayer on the SiO_2_ substrate was followed by the plasma etching process of PS monolayer using a planar geometry inductively coupled plasma reactive ion etching (ICP-RIE) system. The etching process was carried out using oxygen plasma at a bias of 0 V, for a precisely controlled time.

After the etching process, the substrates were moved to the deposition chamber, where the deposition of a 30 nm thick AlO*_x_* buffer film and a 0.9 nm thick Fe catalyst was carried out, using the radio frequency (RF) magnetron sputtering method. The deposition chamber was equipped with an Al_2_O_3_ target and Fe target, both of 99.99% purity and 2 inches diameter, placed horizontally on the top of the vacuum chamber. The deposition took place in the argon (Ar) gas atmosphere at the working pressure of 0.8 Pa. The details were described before in [59]. After the catalyst deposition samples were sonicated in pure ethanol, in order to remove the PS monolayer from the surface, before the process of CNT growth.

The synthesis of CNTs was carried out using a thermal catalytic CVD (CCVD) method on the prepared samples with pristine catalyst. The annealing process was conducted for 3.5 min in the quartz tube furnace (2 inches diameter) heated to 730 °C in a vacuum atmosphere. After that, the CNT growth process was carried out at the same temperature, in the 10 sccm acetylene (C_2_H_2_) gas flow, at a pressure of 60 Pa, for a precisely controlled time. The details were previously described in [60].

The morphology and structure of CNT forest patterns were determined by a field emission scanning electron microscope (FE-SEM) SU8020 (Hitachi High-Tech Corp., Tokyo, Japan). The reflectance of CNT forest fishnet structures in the ultraviolet-visible (UV-Vis) region was measured by the UV-Vis spectrophotometer U3900 (Hitachi High-Tech Corp., Tokyo, Japan). For total reflectance measurements, the incident angle was 10°, while diffuse reflectance was measured at 0° incidence. For the structural and quality analysis of CNT forests, a LabRAM HR-800 micro-Raman (Horiba Ltd., Kyoto, Japan) was used, with a laser excitation wavelength of 532.08 nm. 

## 3. Results

**Figure 2:**
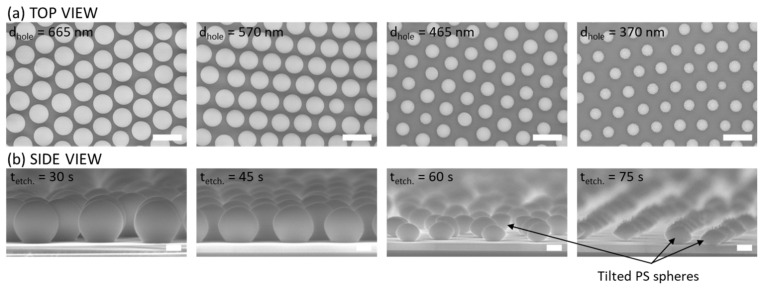
(**a**) Top-view and (**b**) side-view SEM images of self-organized PS monolayer after the ICP-RIE plasma etching treatment. The diameter of PS nanobeads of 665, 570, 465, and 370 nm was obtained for 30, 45, 60, and 75 s etching time, respectively. Scale bars are 1 µm for top-view images, and 200 nm for side-view images.

Figure 2a,b show top-view and side-view SEM images of self-organized PS nanobead monolayers on the surface of SiO_2_, after the oxygen plasma etching, before the catalyst deposition. As can be seen, by the precise control of the etching time of 30, 45, 60 and 75 s, high uniformity of sizes of nanospheres was achieved, resulting in the diameters of 665 ± 16 nm, 570 ± 11 nm, 465 ± 17 nm and 370 ± 20 nm, respectively. Moreover, as compared to the pristine monolayer, the arrangement of the nanospheres after the etching was generally maintained for different diameters of beads, leading to the same distance between centers of spheres (pitch) and keeping the same lattice parameters, which is crucial for the precise fabrication of fishnet structures. However, as can be seen in Figure 2, the decrease in the size of nanobeads might cause some small position changes of nanobeads, due to their very small size, which can potentially introduce some lattice defects to the fishnets. To reduce the possibility of such occurrence, the decreased etching speed, controlled by the pressure or power during the process, is a good solution. The nanobeads shown in Figure 2 were used as shadow masks during the catalyst sputtering process, which allowed for the fishnet structure of AlO*_x_*/Fe film. After the removal of the nanospheres by sonication in ethanol, CNTs were synthesized by the CVD method. 

It should be noticed that the reversal process to obtain CNT pillars can also be achieved using this method, just by changing the order of the fabrication, e.g., if the fabrication of PS monolayer is carried out on the prepared catalyst, the etching process shrinks the nanobeads and also removes the catalyst from the substrate, leaving the pristine catalyst under the PS spheres. Moreover, the control of the distance between the centers of PS nanobeads can be controlled by the initial size of PS spheres.

**Figure 3:**
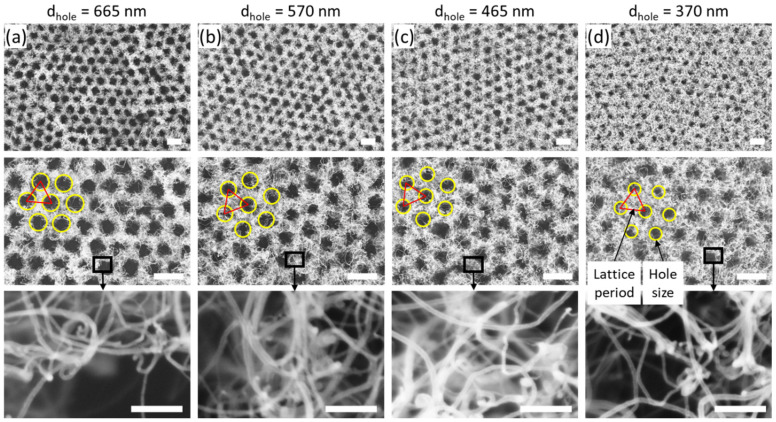
CNT forest fishnet metamaterial structure with (**a**) 665 nm, (**b**) 570 nm, (**c**) 465 nm, and (**d**) 370 nm hole diameters. The constant lattice (pitch) distance and variable size of holes were marked. Scale bars are 1 µm for top and middle row, and 100 nm for bottom row.

Figure 3 shows SEM images of CNT forest fishnet structures after the CVD synthesis process. Large-scale high-quality fishnets were obtained, with different sizes of holes and the same lattice constant of 800 nm (the initial size of PS spheres). As compared to the pristine PS nanobead monolayers, geometrical parameters, such as the holes size and lattice structure, were well preserved after the CNT forest growth. High magnification images in the top wall area are also shown in the bottom row. With the decrease in the hole size, a larger number of thicker MWNTs can be observed, while the number of thin CNTs decreases systematically. Despite a very good structure of CNT fishnets, some minor defects were also observed (Appendix A—Figure A1). First, the PS monolayer self-assembly caused the formation of a multi-domain structure with uniform regions of the different orientations of PS nanobeads. Because of such structure, borders between domains were formed, and after the CNT growth, a slightly increased thickness of walls was observed in the beforementioned regions. Second, the inhomogeneity of the monolayer formation process on the water–air interface was also observed, which included the formation of the second layer on the top of the monolayer and the existence of low-density regions with holes in the PS self-arranged structure. While the second layer resulted in the formation of larger holes, unoccupied by CNTs, low-density regions resulted in the growth of a CNT forest without the fishnet pattern. Third, due to the inhomogeneity of the PS size in the commercially available product, some additional minor defects appeared, such as the change of the size of individual holes in fishnets. Finally, due to the nature of the CNT growth, the entanglement of CNTs could result in partial coverage of the hole at the top region of fishnet structures. To alleviate these issues, in the future, an additional investigation, including the formation of PS monolayer to enhance the self-organization into homogenous monolayers with fewer defects, and the optimization of the hole to CNT area ratio for the precise control of the various properties in CNT forest fishnets, are necessary.

**Figure 4:**
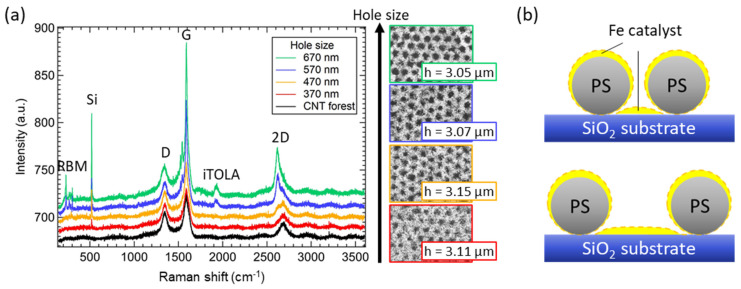
(**a**) Raman spectra of non-patterned CNT forest and CNT forest fishnet structures for different size of holes and similar height of CNTs. (**b**) Schematic representation of the Fe catalyst uniformity during the sputtering process that resulted in the change of the morphology of CNTs in fishnet metamaterials.

To investigate the morphology of CNTs, in Figure 4a, the Raman spectra of the bulk CNT forest and CNT forest fishnets patterns are shown. With the increase in the hole size and thus, the decrease in the fishnet wall thickness, a change of the CNT structure was observed, despite the similar height of CNTs between 3.05 and 3.15 µm. For the bulk CNT forest, the typical spectra of MWNTs were observed, including D, G, and 2D peaks, however without any peaks that could be associated with SWNTs. This structure was expected, as the experimental parameters of CNT synthesis were chosen in order to obtain MWNTs. On the other hand, while CNT fishnets with a hole size of 370 nm still showed a large number of MWNTs, a further increase in the hole size resulted in the gradual change of the structure from MWNTs to SWNTs. For a hole size of 465 nm, the split of the 2D peak was observed and related to the higher ratio of SWNTs. Furthermore, a higher G/D peak intensity ratio was noted. With the further increase in the hole size to 570 nm, the radial breathing mode (RBM) peak and iTOLA peak appeared in the spectrum. The RBM peak is an indicator of SWNTs and was associated with the bond-stretching phonons for which all the carbon atoms move coherently in the radial direction. The high-frequency iTOLA peak is a combination of two phonons, one from the in-plane transverse optical branch (iTO), while the second phonon is related to the longitudinal acoustic branch (LA). The iTOLA peak observation is related to the dispersion of phonon branches in low defective graphite, indicating at the same time a high number of high-quality SWNTs. Furthermore, the intensity ratio of G/D peaks was also increased, confirming that the Raman spectra started to be dominated by SWNTs. Identical peaks with increased intensity were also observed for 665 nm holes. 

The explanation behind the change of the CNT structure from MWNTs to SWNTs was associated with the deposition parameters of catalyst film. In Figure 4b, the small diameter of nanobeads (370 nm holes) allowed for the homogeneous deposition of a uniform film on the SiO_2_ surface between them, because of the small influence of the shading effect. Using the same deposition parameters, with the decrease in the distance between nanospheres, the PS nanobeads cast a larger shadow on the surface, increasing at the same time the area covered with thinner catalyst. This decrease in catalyst thickness resulted in a large number of SWNTs, which also affected the ratio of SWNTs and MWNTs and was clearly visible in the Raman spectra in Figure 4a. Similar measurements of Raman spectra were carried out for the different degrees of self-organization (densities of holes) and are shown in Appendix B. 

**Figure 5:**
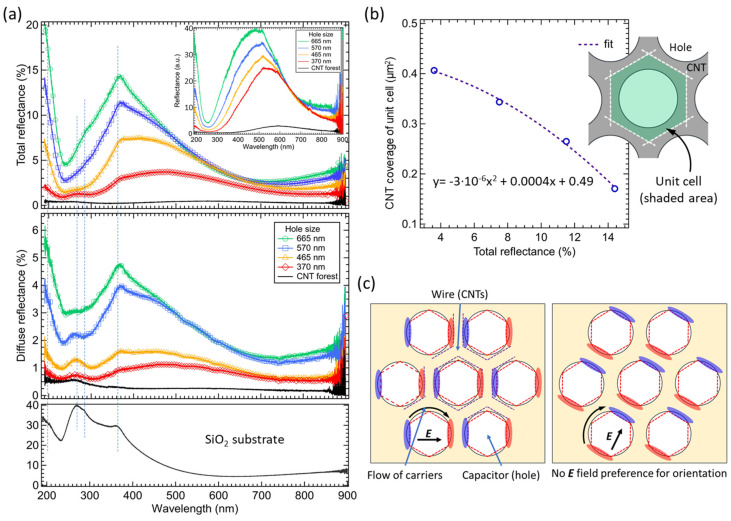
(**a**) Total and diffuse reflectance spectra of CNT fishnet structures with different hole sizes. The spectra of the bare SiO_2_ are included for the extraction of substrate peaks. The inset shows the CNT reflectance spectra with SiO_2_ used as a reference in measurements. (**b**) The total reflectance vs. CNT coverage area of fishnet unit cell. (**c**) The model of the capacitance and inductance parts of the fishnet structure with circulating current due to the orientation of external **E** field vector. Shaded blue and red areas are related to charge distribution of electrons and holes in the fishnet.

The investigation of the optical properties of CNT forest fishnets and non-patterned CNTs was carried out based on the obtained results of the total and diffuse reflectance spectra, as shown in Figure 5a. The SiO_2_ reflectance was measured to identify peaks associated with the substrate. As it can be seen, two main effects of the hole size variation were observed in both spectra. The first one is the increase in the intensity of the reflectance spectra with the increase in the fishnet hole size. Second, a blueshift of the broadband peak was observed from about 500 nm to about 400 nm, for a hole size of 370 nm and 665 nm, respectively.

The change of the intensity of the total reflectance spectra was related to the change of the absorption area with the introduction of holes in fishnets. With larger holes, the area not covered with CNTs (bare substrate area) was increased, which naturally increased the reflectance from the entire fishnet structure. In order to fully investigate possible interactions of CNT fishnets with the EM waves, the change of the reflectance intensity at the peak position vs. the CNT coverage area of the unit cell was investigated and showed a nonlinear characteristic (Figure 5b). In other words, if no additional interaction of the CNT structure with the EM wave occurred, the change of the absorption area should result only in a linear change of the intensity of reflectance. However, as noted, the nonlinear change of intensity was observed as a result of additional interactions between the CNT forest and the incident EM wave, which caused additional light absorption in CNT fishnets. 

To confirm the nonlinear reflectance results, spectra with the SiO_2_ substrate as the reference were also measured (Figure 5a—inset) to show only the response from CNTs. The change of the reflectance intensity and the blueshift of the broadband peak position were observed also in this case. Interestingly, the shift of the resonance peak for the diameter change from 370 nm to 665 nm was observed between 550 nm and 460 nm wavelength, respectively, which is different from the previous values (450 nm and 360 nm). It was assumed that, since the effect of the SiO_2_ substrate was entirely removed from the spectra, the obtained position of reflectance peaks was only CNT response, without substrate peak superposition.

In diffuse reflectance spectra (Figure 5a), the increase in the hole diameter resulted in the increase in the reflectance values with a relatively big step between the 465 nm and 570 nm holes. This more significant increase in the reflectance can be explained by the change of the morphology of CNTs, from MWNTs to SWNTs, as observed in the Raman spectra (Figure 4), in which the change of hole size from 465 nm to 570 nm, resulted in the excitation of additional peaks (RBM, iTOLA). SWNTs usually produce stronger entanglement of the top layer of the CNT forest that leads to increased diffuse reflectance. 

Generally, the nonlinear reflectance results can be associated with two diverse effects, the morphology of a CNT forest and the geometry of the fishnet structure. In CNT forests, the degree of changes of the reflectance depends mostly on the height of CNTs, the density and the alignment of CNTs in the forest, and the type of CNTs (MWNTs, SWNTs) [59]. As mentioned before, the height of CNT forest fishnets was similar, to avoid its influence on the results; thus, it can be omitted. The density and alignment could result in the change of reflectance values, due to a gradual increase in the SWNT ratio with an increase in the hole size. However, since the walls of fishnet structures are relatively thin and high density or alignment is difficult to obtain in such case, it was assumed that the density or alignment discrepancies would not result in such large nonlinear changes of reflectance, as in Figure 5a. At the same time, the morphology of CNT has a small influence on the results, although it changes the ratio of diffuse reflectance in the total reflectance spectra. 

Regarding the blueshift of the broadband reflectance peak that was observed in total and diffuse spectra, this was caused by the change of the wire width in the fishnet structure. With the increase in the hole size, the thickness of the CNT walls (wires in fishnet) was reduced. By decreasing the width of the wire (larger hole size), the inductance of the circuit of fishnet metamaterial structure increases, allowing for a stronger magnetic resonance to be generated, which resulted in the change of the resonant frequency of CNT structures and the shift towards lower frequencies. At the same time, since the change of the width of the wires and the diameter of the holes cannot be treated separately, with the same variation of the hole size from 370 nm to 665 nm, the capacitance in the fishnet decreased, causing the shift towards higher frequencies that dominates over the decrease in the inductance, as shown in Figure 5a.

To further explain the nonlinear behavior, a simplified model of the LC resonance in CNT forest metamaterial fishnets is shown in Figure 5c. The geometry of the CNT fishnets allows the formation of different areas that can be identified as wires and capacitors. With the interaction of the electric field (***E***) vector with the holes (capacitor gaps), the current is induced in the structure, and the charge transfer can be achieved by direct contact or tunneling effect in CNTs [26]. Furthermore, since the structure is isotropic, due to the circular design of the fishnet holes, there is no preference for the orientation towards the ***E*** field. It means that any orientation of the ***E*** field can enhance the resonance behavior, supporting the flow of the circulating current and allowing for the formation of electric dipoles with electrons and holes grouped on different sides of capacitors in the fishnets. This leads to areas with larger and smaller saturation of carriers and since additional electron–hole pairs can be generated in CNTs under light irradiation in smaller saturation areas, additional absorption of light can be observed, e.g., as the nonlinear reflectance dependence shown in Figure 5b.

## 4. Conclusions

In this work, the fabrication of CNT forest fishnet metamaterials with various geometry using self-organized PS nanospheres was confirmed, and the optical properties of the obtained structures were investigated. In the fabrication process, high-quality large-scale CNT forest fishnets were successfully fabricated with 800 nm pitch and a different size of the holes (370, 465, 570 and 665 nm), which were controlled by the initial size of PS nanobeads and the etching time required to shrink the PS spheres in the monolayer, respectively.

In the Raman spectra, the increase in the hole size (decrease in the wall thickness) caused the change of the morphology of the CNT forest from multi-walled CNTs to single-walled CNTs, due to the shading effect of the PS nanobeads during the catalyst deposition. A larger shading effect caused by the smaller distance between nanobeads resulted in a thinner catalyst film and the growth of SWNTs.

In the UV-Vis reflectance spectra, the increase in the hole size from 370 nm to 665 nm in the CNT fishnets resulted in the blueshift of the observed broadband reflectance peak and the nonlinear change of the reflectance intensity. Both of these effects (nonlinear light absorption and change of resonance frequency) were associated with interactions of light with CNT patterns, due to the inductance and capacitance changes caused by the fishnet geometry variation. The isotropic fishnet structure with no preference for the orientation towards the ***E*** field allowed for enhanced current flow and rotation of electric dipoles in fishnets, supporting additional nonlinear absorption of light.

The presented results show large potential for future energy harvesting and storage devices, solar cells, and various optoelectronic devices, such as neuromorphic networks, or light emitters.

## Figures and Tables

**Figure A1:**
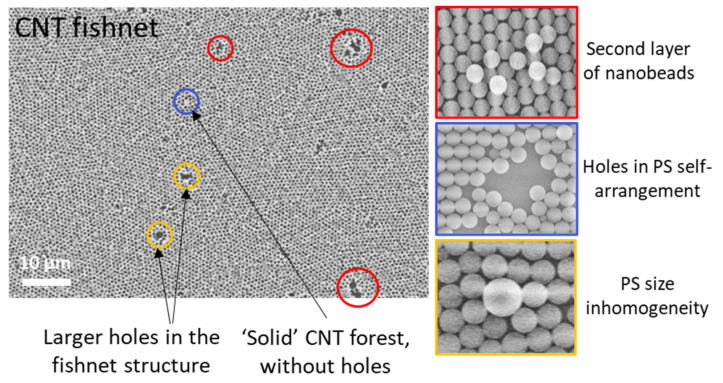
Defects in the CNT forest fishnet metamateials due to the preparation of the PS monoloayer film.

**Figure A2:**
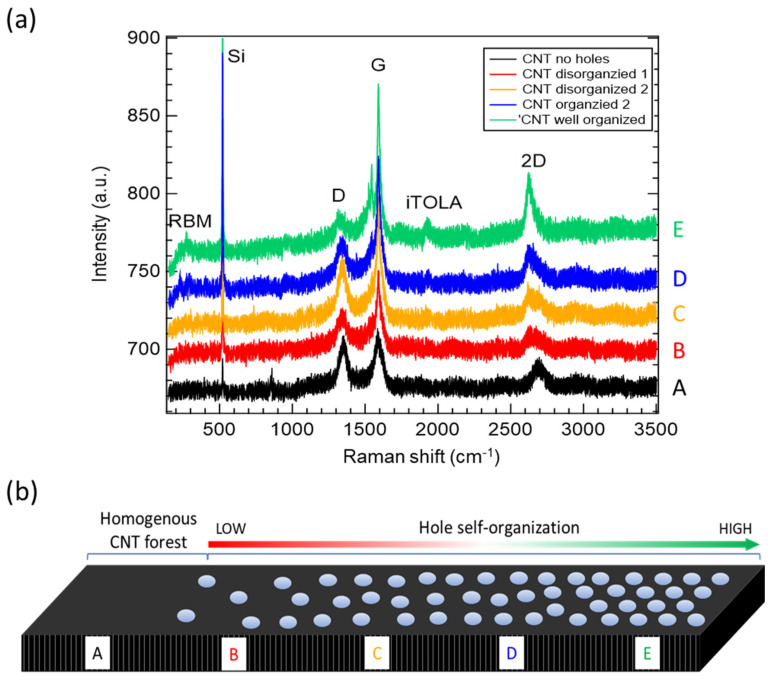
(**a**) Raman spectra of CNT forest with different degree of fishnet structure organization. (**b**) Schematic of the change of the self-organization of the CNT forest.

## Data Availability

The data that supports the findings of this study are available from the corresponding author upon reasonable request.

## Appendix A

The defects of the PS monolayer self-assembly are caused by the second layer of PS nanobeads, holes in the self-arrangement, and the size inhomogeneity of PS spheres. The second layer caused the formation of large holes during CNT growth. Unoccupied areas resulted in the growth of solid CNT forest. The size inhomogeneity caused the larger diameter of holes in fishnets.

## Appendix B

Figure A2a shows the Raman spectra for the CNT forest taken from the same sample with different organizations of the fishnet structure. As shown in the schematic in Figure A2b, the gradual transformation from homogenous CNT forest [A] into highly organized fishnet metamaterial [E] caused the change of the CNT morphology. With the increase in the number of holes and their organization, the intensity of G-peak gradually increased, while the 2D peak also showed a shift related to the increased CNT quality, originating from SWNTs. The iTOLA and RBM peaks also became more visible with the increase in the degree of fishnet organization. Due to smaller CNT coverage in the measurement area, the Si substrate peaks also become more visible with increased self-organization.
